# Supplementary material for: Pyruvate Dehydrogenase Kinase 1 inhibition mediated oxidative phosphorylation enhancement in cartilage promotes osteoarthritis progression
Source: BMC Musculoskelet Disord. 2023 Jul 20;24:597. doi: 10.1186/s12891-023-06585-6 (PMC10357736; doi:10.1186/s12891-023-06585-6)
Supplement: Supplementary file 1 — Additional file 1. [file 12891_2023_6585_MOESM1_ESM.zip › MF.Gsea.1653627316242/GOMF_VITAMIN_TRANSMEMBRANE_TRANSPORTER_ACTIVITY.html]

Details for gene set GOMF\_VITAMIN\_TRANSMEMBRANE\_TRANSPORTER\_ACTIVITY[GSEA]

|  || Dataset | 55457\_collapsed\_to\_symbols.cls.cls #OA\_versus\_normal.cls.cls #OA\_versus\_normal\_repos |
| Phenotype | cls.cls#OA\_versus\_normal\_repos |
| Upregulated in class | normal |
| GeneSet | GOMF\_VITAMIN\_TRANSMEMBRANE\_TRANSPORTER\_ACTIVITY |
| Enrichment Score (ES) | -0.486379 |
| Normalized Enrichment Score (NES) | -1.579913 |
| Nominal p-value | 0.015075377 |
| FDR q-value | 0.15367313 |
| FWER p-Value | 0.998 |
Table: GSEA Results Summary

  

Fig 1: Enrichment plot: GOMF\_VITAMIN\_TRANSMEMBRANE\_TRANSPORTER\_ACTIVITY      
 Profile of the Running ES Score & Positions of GeneSet Members on the Rank Ordered List

  

| SYMBOL | TITLE | RANK IN GENE LIST | RANK METRIC SCORE | RUNNING ES | CORE ENRICHMENT || 1 | SLC2A10 | solute carrier family 2 member 10 [Source:HGNC Symbol;Acc:HGNC:13444] | 874 | 0.550 | 0.0049 | No |
| 2 | SLC2A2 | solute carrier family 2 member 2 [Source:HGNC Symbol;Acc:HGNC:11006] | 3168 | 0.234 | -0.1422 | No |
| 3 | SLC2A6 | solute carrier family 2 member 6 [Source:HGNC Symbol;Acc:HGNC:11011] | 4449 | 0.117 | -0.2261 | No |
| 4 | RBP4 | retinol binding protein 4 [Source:HGNC Symbol;Acc:HGNC:9922] | 5072 | 0.067 | -0.2656 | No |
| 5 | PDPN | podoplanin [Source:HGNC Symbol;Acc:HGNC:29602] | 5594 | 0.024 | -0.3028 | No |
| 6 | GC | GC vitamin D binding protein [Source:HGNC Symbol;Acc:HGNC:4187] | 5856 | 0.005 | -0.3225 | No |
| 7 | SLC2A8 | solute carrier family 2 member 8 [Source:HGNC Symbol;Acc:HGNC:13812] | 6137 | -0.015 | -0.3422 | No |
| 8 | SLC2A1 | solute carrier family 2 member 1 [Source:HGNC Symbol;Acc:HGNC:11005] | 7089 | -0.087 | -0.4046 | No |
| 9 | STRA6 | signaling receptor and transporter of retinol STRA6 [Source:HGNC Symbol;Acc:HGNC:30650] | 7107 | -0.089 | -0.3942 | No |
| 10 | SLC52A1 | solute carrier family 52 member 1 [Source:HGNC Symbol;Acc:HGNC:30225] | 7145 | -0.092 | -0.3849 | No |
| 11 | ABCG2 | ATP binding cassette subfamily G member 2 (Junior blood group) [Source:HGNC Symbol;Acc:HGNC:74] | 7473 | -0.116 | -0.3949 | No |
| 12 | ABCD4 | ATP binding cassette subfamily D member 4 [Source:HGNC Symbol;Acc:HGNC:68] | 8652 | -0.205 | -0.4593 | Yes |
| 13 | SLC23A2 | solute carrier family 23 member 2 [Source:HGNC Symbol;Acc:HGNC:10973] | 8732 | -0.211 | -0.4374 | Yes |
| 14 | SLC44A4 | solute carrier family 44 member 4 [Source:HGNC Symbol;Acc:HGNC:13941] | 8853 | -0.221 | -0.4175 | Yes |
| 15 | SLC25A32 | solute carrier family 25 member 32 [Source:HGNC Symbol;Acc:HGNC:29683] | 9170 | -0.246 | -0.4095 | Yes |
| 16 | C2orf83 | chromosome 2 open reading frame 83 [Source:HGNC Symbol;Acc:HGNC:25344] | 9279 | -0.254 | -0.3842 | Yes |
| 17 | SLC19A1 | solute carrier family 19 member 1 [Source:HGNC Symbol;Acc:HGNC:10937] | 9931 | -0.315 | -0.3931 | Yes |
| 18 | SLC19A3 | solute carrier family 19 member 3 [Source:HGNC Symbol;Acc:HGNC:16266] | 10126 | -0.334 | -0.3640 | Yes |
| 19 | ABCA4 | ATP binding cassette subfamily A member 4 [Source:HGNC Symbol;Acc:HGNC:34] | 10554 | -0.379 | -0.3471 | Yes |
| 20 | SLC22A14 | solute carrier family 22 member 14 [Source:HGNC Symbol;Acc:HGNC:8495] | 11139 | -0.448 | -0.3331 | Yes |
| 21 | ABCC1 | ATP binding cassette subfamily C member 1 [Source:HGNC Symbol;Acc:HGNC:51] | 11462 | -0.497 | -0.2923 | Yes |
| 22 | SLC5A6 | solute carrier family 5 member 6 [Source:HGNC Symbol;Acc:HGNC:11041] | 11809 | -0.551 | -0.2461 | Yes |
| 23 | SLC19A2 | solute carrier family 19 member 2 [Source:HGNC Symbol;Acc:HGNC:10938] | 12600 | -0.798 | -0.2017 | Yes |
| 24 | SLC2A3 | solute carrier family 2 member 3 [Source:HGNC Symbol;Acc:HGNC:11007] | 12604 | -0.800 | -0.0961 | Yes |
| 25 | SLC52A2 | solute carrier family 52 member 2 [Source:HGNC Symbol;Acc:HGNC:30224] | 12735 | -0.899 | 0.0128 | Yes |
Table: GSEA details [plain text format]

  

Fig 2: GOMF\_VITAMIN\_TRANSMEMBRANE\_TRANSPORTER\_ACTIVITY      
 Blue-Pink O' Gram in the Space of the Analyzed GeneSet

  

Fig 3: GOMF\_VITAMIN\_TRANSMEMBRANE\_TRANSPORTER\_ACTIVITY: Random ES distribution      
 Gene set null distribution of ES for **GOMF\_VITAMIN\_TRANSMEMBRANE\_TRANSPORTER\_ACTIVITY**
